# Genome-wide Identification of IRF1 Binding Sites Reveals Extensive Occupancy at Cell Death Associated Genes

**DOI:** 10.4172/2157-2518.S6-009

**Published:** 2013

**Authors:** Alessandro Rettino, Nicole M Clarke

**Affiliations:** School of Pharmacy, Centre for Biomolecular Sciences, University of Nottingham, Nottingham, NG7 2RD, UK

**Keywords:** Interferon regulatory factor 1, ChIP-seq, Cell death of associated genes

## Abstract

IRF1 is a transcription factor involved in interferon signaling and has been shown to harbor tumor suppressor activity. In order to comprehensively identify pathways regulated by IRF1, we used chromatin immunoprecipitation followed by massive-parallel sequencing (ChIP-seq) to evaluate the gene targets of IRF1 genome-wide. We identified 17,416 total binding events in breast cancer cells. Functional categorization of the binding sites after IFN-gamma (interferon-gamma) treatment determined that ‘apoptosis’ or ‘cell death’ is the most enriched target process. Motif discovery analysis of the chromosomal regions bound by IRF1 identified a number of unique motifs correlated with apoptosis, DNA damage and immune processes. Analysis of GEO transcriptome data from IRF1-transduced cells or IFN-gamma treated fibroblasts indicates that IRF1-bound targets in IFN-treated cells are associated with a positive transcriptional response. Many of the enriched target genes from the expression analysis are associated with apoptosis. Importantly, this data indicates that a significant function of IRF1 is the regulation of anti-cancer apoptotic pathways and this reinforces IRF1’s role as a tumor suppressor.

## Introduction

Interferon regulatory factor 1 (IRF1) was the first interferon regulatory factor family protein identified (family members consist of IRF1-9) [1]. All of the family members share homology in their DNA binding domains, which contain a tryptophan repeat consisting of five tryptophans [2]. It was first shown to bind to upstream sequences of the interferon-beta gene [3]. In vitro DNA selection studies identified a consensus sequence termed the IRF-E (G(A)AAAG/CT/CGAAAG/CT/C) [4] which is very similar to the ISRE found in interferon stimulated genes (A/GNGAAANNGAAACT). IRF1 levels are low in unstimulated cells but it is induced by different cytokines, IFNs (α, β, γ), TNF-α, IL-1, IL-6, and by viral infection [5,6]. Structure studies of IRF1 bound to DNA determined that IRF1 contacts the DNA through three of the conserved tryptophan residues. The authors identified a core DNA sequence for IRF1 binding which is GAAA and suggested that IRF1 may bind target genes through repeats of the core GAAA sequence [2].

IRF1 is involved in the development and differentiation of immune cells. Using gene knockouts, IRF1 was shown to be important for Natural Killer (NK) cell development. IL-15 is a gene target of IRF1 and the absence of IRF1 results in a lack of IL-15 production important in the microenvironment supporting the maturation of NK cells. Specifically IRF1 −/− mice showed dramatically reduced NK1.1+TCR α/β and decreased NK-cell-mediated cytolytic activity [7-9]. IRF1 null mice also show increased susceptibility to infection by specific pathogens such as *Leishmania major* which is thought to be due to a defective T helper 1 (Th1) response. T cells from IRF1 mice fail to mount a Th1 response and are skewed toward a Th2 response with impaired production of IL-12 [10,11]. Thus, IRF1 is also important for the differentiation of T helper cells.

IRF1 has also been shown to be involved in a number of other diverse cellular processes such as DNA damage, cell cycle and oncogenesis. IRF1 −/− MEFs are deficient in their ability to undergo DNA damage-induced cell cycle arrest similar to MEFs lacking the tumor suppressor, p53. Also, IRF1 is important for the DNA damage-induced apoptosis of mitogenically activated mature T lymphocytes [12,13] These studies suggested that IRF1 might harbor tumor suppressor activity. This was reinforced by studies showing that IRF1 is able to revert the transformed phenotype of oncogene-transformed cells and IRF1 −/− MEFs are susceptible to transformation by the activated form of the c-H-ras gene [14,15]. Further studies in IRF1 −/− mice demonstrated that IRF1 is a tumor susceptibility gene. Crossing IRF1 −/− mice with p53 −/− mice resulted in a large increase in the number of tumors, as well as, the tumor spectrum of p53−/− mice. These data indicated that loss of IRF1 exacerbates previous tumor predisposition and most likely regulates tumor suppressor pathways independent of p53 [16]. In addition, another IRF1 −/− murine model revealed that loss of IRF1 results in a lymphoproliferative disease that mimics ALCL [17].

A number of targets for IRF1 have been identified in single gene studies. IL-15 (7), IL-12 (10,11,18), MHC class II transactivator CIITA, TAP-1 and LMP-2 are IRF1 targets involved in immune responses [18-20]. IRF-1 is thought to target p21 in and caspase-1 in DNA damaged cells. Other single gene studies have identified apoptotic gene targets such as caspase-8, TRAIL, PUMA and XAF1 [21-24]. Recent studies looked at IRF1 targets in a high-throughput manner. ChIP-chip analysis of IRF1 targets in IFN-gamma treated breast cancer cells identified a number of new targets in the DNA damage response including BRIP1 [25]. A Chip-sequencing study of IRF1 in primary human monocytes also identified 52 bound regions associated with target genes mainly mapping to immune responses such as AIM2 and IFIT3 and a new 18-bp binding motif enriched in target sequences [26].

We undertook a high-throughput ChIP-sequencing study of IRF1 chromatin bound regions in unstimulated and IFN-gamma stimulated breast cancer cells in order to increase our understanding of pathways regulated by IRF1 in cells. Gene ontology analysis identified cell death as a major target pathway of IRF1 in IFN stimulated cells. De novo motif discovery identified a set of DNA sequences enriched in specific functional pathways such as DNA damage or antigen presentation. Transcriptomic analysis suggests that binding of IRF1 to target regions results in up-regulation of gene expression in IFN-gamma stimulated cells.

## Material and Methods

### Chromatin immunoprecipiation and ChIP-sequencing

Chromatin immunoprecipitation was performed on H3396 breast cancer cells as previously described. H3396 cells were maintained in culture and treated with 1000 units/mL of IFN-gamma for three hours as previously described [25]. Chromatin from three samples for each condition were immunoprecipitated using the IRF1 sc-497 antibody (Santa Cruz Biotechnology). The ChIP-seq library was prepared using the Illumina ChIP sample preparation kit following the manufacturer’s protocol. ChIP-seq libraries were sequenced sequenced on the Genome Analyser II-x (GAIIx) machine, processed and analyzed using the Illumina Genome Analyser (GA) Pipeline software (version 1.5.1) for base calling and sequence alignment. All sequencing and data processing was performed at Source Bioscience.

Briefly, sequence data generated by the Illumina GAIIx platform are outputted in FASTQ format. FASTQ allowed quality scores to be assigned to each base in the read as Phred scores. An additional quality assessment measure was performed to give each read an average quality read (passing a purity filter). Reads were aligned to the hg18 genome build using the GERALD program. Output read alignments were then analyzed using the Illumina Genome Studio software, where regions and peaks of high binding density for IRF1 were identified. Region detection consisted of three sub-routines, moving sum determination, thresholding and region identification. Moving sum determination took a sequence of reads, each specified by a starting location and a read length, and a window size W. An array of summed base counts was returned. Thresholding then took this array, and a threshold value T, to filter the array so that only values above the given threshold value were used. A region was then defined as a maximal run of summed base counts above this threshold. Once regions were identified, peaks within these regions were determined.

Detailed explanations and algorithms used by the GenomeStudio ChIP Sequencing Module can be found at: http://support.illumina.com/array/array_software/gnomestudio/documentation.ilmn.

### Motif discovery

Peak regions were used as seeds for motif discovery. Bed files of chromosomal peak regions were converted to FASTA format and submitted to MEME-ChIP. MEME-ChIP searches for motif enrichment in input sequences and returns motifs as position-dependent letter-probability matrices [27].

### Re-analysis of GEO expression data

Expression data contained in the Gene Expression Omnibus were submitted to GEO2R for calculation of differential expression between two conditions. Raw GEO datasets were assigned to control or treated groups. Data submitted is compared using GEOquery and limma ((Linear Models for Microarray Analysis) R packages [28,29]. The difference in the group-wise expression change between control and IFN-stimulated groups was evaluated with Student’s t test.

### Annotation and gene ontology analysis (GO)

Annotation of peak regions was performed by submitting a bed file of chromosomal coordinates to GPAT. Genes were annotated based on distance from the transcriptional start site of coding regions (within 10 kb of a TSS), in addition any genes directly mapped to a chromosomal coordinate were annotated. GO was performed on gene data sets from the ChIP-sequencing annotated gene list by submitting lists to DAVID for identification of overrepresented biological processes. Pathway analysis was performed using GREAT by submitting bed files of ChIP-seq peak regions. Gene ontologies associated with enriched DNA motifs from MEME-ChIP were derived from GOMO [30-32].

## Results

**Figure 1:**
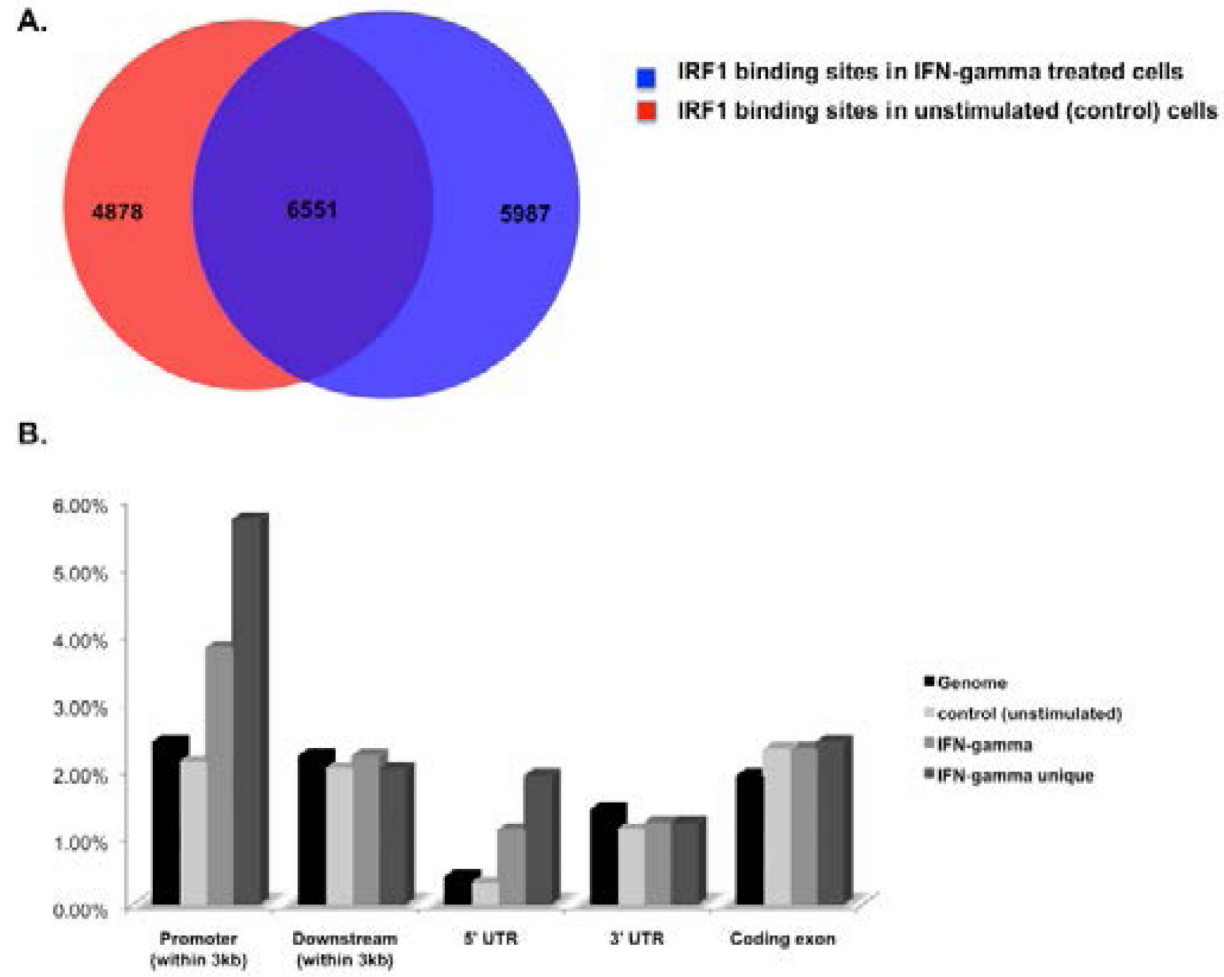
ChIP-sequencing identifies IRF1 binding sites in H3396 breast cancer cells A. Shown are the number of peak regions identified in control (unstimulated) and IFN-gamma stimulated cells B. Positional chromosomal annotation (CEAS) of IRF1 bound peak regions represented as percent coverage. Shown is the set coverage for control, IFN-gamma unique (only found in IFN stimulated cells) and IFN-gamma total binding sites relative to the genome.

In order to identify new IRF1 gene targets genome-wide we performed a ChIP-sequencing study in human breast cancer cells, H3396. Cells were left unstimulated or stimulated with IFN-gamma for 3 hours. Chromatin immunoprecipitated with IRF1 antibody (3 independent samples for each condition) was sequenced and analyzed using Illumina software. There was total of 36 million reads generated for both control and IFN-gamma stimulated chip samples. Reads were quality filtered and then aligned to the hg18 human genome build. Peak regions for each condition (control or IFN-treated) were identified by moving sum determination followed by thresholding (Illumina Genome Studio package). A total of 17, 416 peak regions were identified, which corresponded to 11, 429 peak regions in control (unstimulated) cells and 12, 538 peak regions in IFN-gamma stimulated cells (Figure 1A). There is a total of 5987 unique IRF1 binding events in IFN-gamma stimulated H3396 cells and 4878 unique IRF1 binding events in unstimulated (control) cells. 6551 IRF1 binding events are shared between the two conditions. A number of these shared binding events had increased IRF1 read counts indicating increased binding of IRF1 to these regions in IFN-stimulated cells. Many of the shared regions contained similar read counts indicating that IRF1 occupancy remains the same even after IFN-gamma stimulation at these regions.

Peak regions were annotated using GPAT [32] within 10 kb (promoter) of a transcriptional start site (TSS). All regions falling within a gene-coding region were also automatically annotated (Supplementary Table 1). Enrichment at specific genomic locations was identified for regions bound in control (unstimulated) or exclusively in IFN-gamma stimulated cells (IFN-gamma unique), as well as the total pool of IFN-gamma bound regions (Figure 1B). Binding of IRF1 in unstimulated cells did not show any enrichment at any particular genomic feature. In contrast location analysis (using CEAS) [33] of total IRF1 binding events in IFN-gamma treated cells or IFN-gamma unique binding events showed a dramatic increase in binding at promoter regions (4% and 6%, respectively compared to 2% in control cells). There was also a dramatic increase in binding to the 5′ UTR regions of genes (1% and 2% respectively, compared to 0.4% in control cells). These data demonstrate that IRF1 is highly recruited to regions directly upstream or downstream of transcriptional start sites in IFN-gamma stimulated cells where it can have a direct effect on transcriptional activity of these genes.

**Figure 2:**
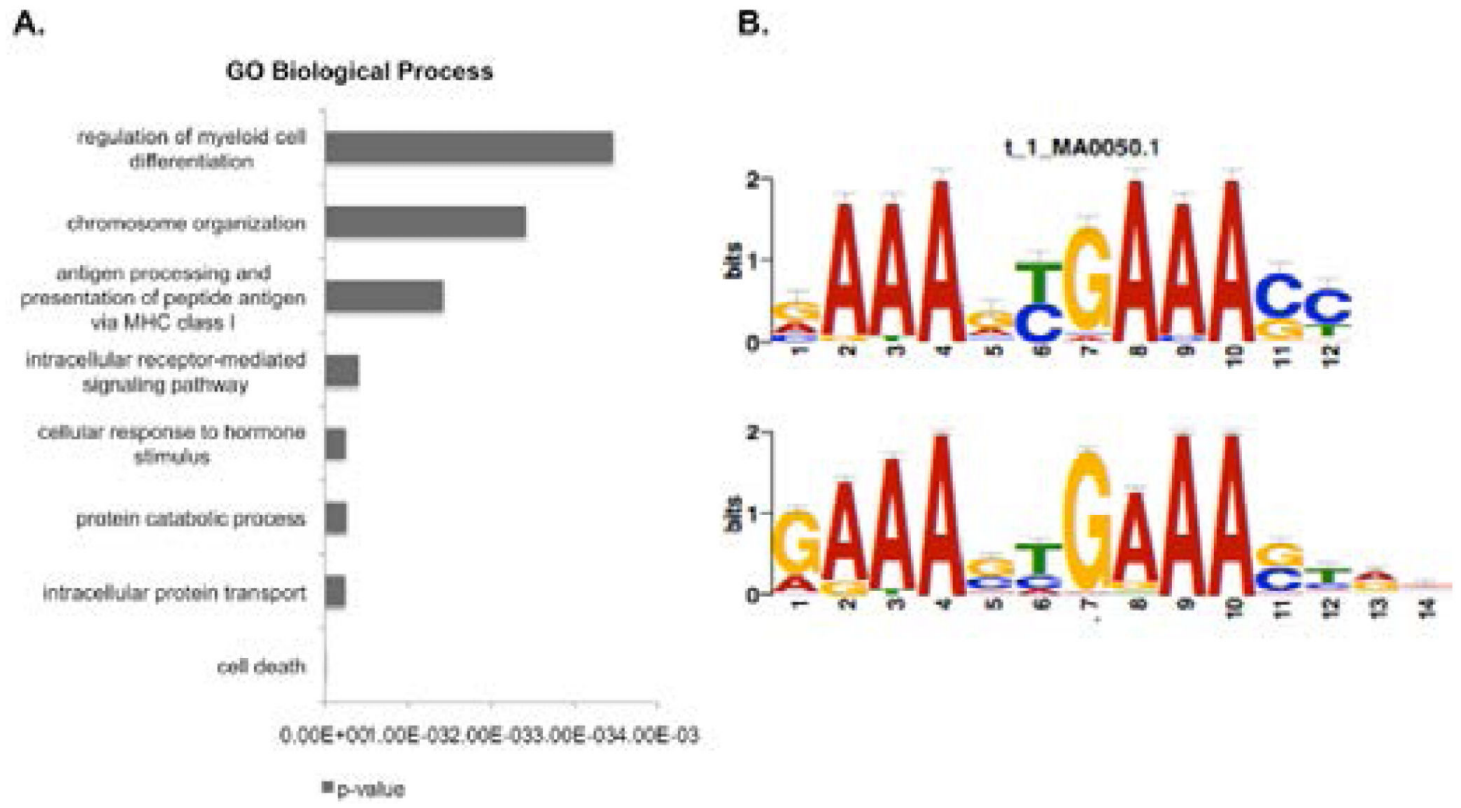
Cell death is the top biological process enriched in genes associated with IRF1 peak regions A. The graph displays significantly enriched biological processes (shown as p-value) based on the gene ontology of genes in close proximity to IRF1 peak regions (within 10 kb upstream of a TSS) from IFN-gamma stimulated cells B. Shown is the logo derived from Motif analysis using MEME-ChIP for annotated genes categorized under cell death versus the consensus motif for IRF1 (e-value, 1.2e-73).

As we were interested in defining IRF1 pathways important for its’ tumor suppressor activity, we performed analysis of enriched functional categories for the annotated genes bound in control (unstimulated) (Supplementary Figure 1) or IFN-gamma stimulated cells using DAVID [30,31]. In IFN treated cells, cell death is the most statistically significant GO biological process, followed by intracellular protein transport (Figure 2A). Cell death genes are also significantly associated with IRF1 bound regions in control (unstimulated) cells. Interestingly, another enriched category is ‘antigen processing and presentation of peptide antigen via MHC class I’. IRF1 is known to target a number of the genes in this pathway and suggests that the ChIP-sequencing was successful in identifying true IRF1 binding events. Another interesting category is ‘regulation of myeloid cell differentiation’. This is in agreement with a previous study of IRF1−/− mice that suggested that IRF1 was important for myelopoiesis [34]. As cell death is the major biological process putatively controlled by IRF1 in IFN-gamma stimulated cells, we wanted to determine if these targets were enriched in the IRF consensus motif. 120 genes were clustered in the cell death category. Motif analysis of the peak regions from these 120 genes using MEME-ChIP identified an enriched motif that matches the IRF1 consensus motif (Figure 2B).

Further pathway analysis (GREAT) [35] was performed on the IFN-stimulated peak region associated genes. Here, the regions were separated into those associated with peaks exclusive to IFN-stimulated cells (IFN-gamma unique) and total IFN-gamma peak regions (which includes regions that are also bound in control cells). Interestingly, the ATR signaling pathway was identified as enriched in total IFN-gamma annotated genes. A previous IRF1 ChIP-chip study identified a number of DNA damage gene targets [25] and this data suggests that IRF1 may regulate other proteins involved in DNA damage signaling in addition to the Fanconi anemia gene, BRIP1. Apoptosis is again a significantly enriched pathway in both gene sets, as well as, interferon signaling pathways (α, β, γ). This is expected as IRF1 is known to be induced by interferons and to regulate interferon-stimulated genes and this indicates that bona fide IRF1 targets are enriched in the peak regions. In the IFN-gamma unique gene set, the extrinsic death receptor pathway is specifically enriched. Among this gene set is caspase 8 and TRAIL, two known targets of IRF1 and indicate that death receptor signaling is specifically regulated by IRF1 in IFN-gamma stimulated cells and not in unstimulated cells.

Next, the distance of peak regions from known TSS’s (transcriptional start sites) were mapped (using GPAT). Peak regions within 10 kb upstream and downstream of the TSS of a gene coding region were binned and the number of binding sites (counts) were mapped against the distance from the TSS (Figure 4A). The data show that in control (unstimulated) cells there is no clustering of IRF1 binding sites and peak regions are spread evenly within 10 kb of a TSS. In contrast, in IFN-gamma stimulated cells IRF1 binding sites are clustered around the TSS (−1kb to +2kb) of genes. The peak associated genes within this cluster were analysed for GO biological processes, which indicated that IRF1 binding sites near the TSS are associated with genes involved in apoptosis, response to virus, immune responses, antigen presentation and JAK-STAT signaling (Figure 4B).

DNA motif analysis of peak regions in control (unstimulated) cells did not produce any significantly enriched motifs. Analysis of the interferon stimulated peak regions (IFN-gamma unique) yielded a number of DNA motifs that slightly differs (at position 5) from the consensus IRF1 binding site in the transcription factor database contained in MEME-ChIP (JASPAR) [27]. Interestingly, the two most significantly enriched motifs from the IFN treated cells were longer in length but maintained a central core which is identical to the consensus originally mapped by in vitro DNA studies, (G(A)AAAG/CT/CGAAAG/CT/C)(Figure 5A and 5B) [4]. In Figure 5A, the motif carries a 5′ extension of 4 A’s and a 3′ extension of 2 A’s. This would yield two binding sites for IRF1 [36]. The second motif in Figure 5B has a 5′ extension of 2 A’s, this would also yield two binding sites for IRF1 whereas the consensus sequence yields one binding site for IRF1. Also, the central NN core from the ChIP-seq enriched motifs indicates that IRF1 prefers a central ‘GTG’ sequence in the IRF-E. This correlates with a recent ChIP-seq study in primary human monocytes which also mapped a longer DNA motif, 18bp in length with a central ‘GTG’ consensus. Other DNA motifs were enriched in the IFN gene sets shown in Supplementary Figure 2. Similar to the apoptotic genes, a motif matching the consensus IRF-E was found. There are two 15bp motifs and one 21bp motif, which yield two IRF1 binding sites each. Lastly, there is one 20bp motif (e-value, 1.1 e-81), which yields three IRF1 binding sites. This is interesting and differs from the ChIP-seq carried out in primary human macrophages that found an 18bp motif yielding two IRF1 binding sites. This motif is found exclusively in IFN-gamma treated cells, where binding of IRF1 is not found in control (unstimulated) cells. Thus, IRF1 seems to only bind this sequence after being induced in IFN-treated cells. This corresponds to a DNA sequence, 5′-AANNGAAAC/G/ATGAAAGTG/AAAA-3′.

In order to correlate binding of IRF1 to a transcriptional output, re-analysis of transcriptomic data in the GEO (Gene Expression Omnibus) was undertaken. Two data sets were analysed using GEO2R (expression analysis tool using R programming) to identify differentially expressed genes. We analyzed the GEO dataset, GSE26817 that contains data from a study on IRF1 transduction in Huh-7 human hepatoma cells [37]. In order to look for differential expression, ratios of IRF1 transduced expression to vector alone (FLuc) were calculated. Values were expressed as Log2 ratio with corresponding p-values, where valid log2 ratios with a p-value ≤ 0.05 were retrieved for further analysis. Next, gene lists were compared to identify genes from the ChIP-seq annotated gene list that were present in the validated expression gene list from GSE26817 (Supplementary Table 2) (IFN-gamma annotated genes) and Supplementary Table 3 (control annotated genes)). Distribution analysis of the corresponding log2ratios for control bound IRF1 targets and IFN bound IRF1 targets were plotted (Figure 6A). The median value for IFN-stimulated targets was higher than control bound targets (0.537 vs. 0.399 (p-value=0.0173)). This suggests that IRF1 binding in IFN-gamma treated cells results in higher expression of putative target genes or an increased likelihood of a positive transcriptional response upon IRF1 binding. Of the IRF1 bound genes found on the GSE26817 array 31 overlapped between the control condition and the IFN-gamma stimulated condition (Figure 6B). When we looked at the percentage up-regulation of these genes there was a definite trend, where genes that are bound by IRF1 in unstimulated cells are less likely to be up-regulated (55%) (Figure 6C). Genes that are shared between the two conditions had an increased number of up-regulated genes (71%). Finally, genes that were uniquely bound by IRF1 in IFN-gamma treated cells had the highest number of up-regulated genes (87.5%). The second dataset tested was GSE3920, which contained data from IFN-gamma treated fibroblast cells [38]. The data was also re-analysed using GEO2R to determine the log2 ratio of IFN-stimulated vs. unstimulated fibroblasts. A total of 215 annotated putative IRF1 gene targets from the ChIP-seq IFN-gamma stimulated peak region list were identified. Again a large proportion of the corresponding genes (a total of 192 out of the 215) were up-regulated in IFN-gamma treated fibroblasts (89.3%) (Supplementary Table 4). We next looked at the GO biological processes of IRF1 bound genes from the Huh-7 transcriptomic experiment. We found a large proportion of the enriched processes involved the immune system such as inflammatory response, innate immune response, JAK-STAT signaling and leukocyte activation. Cell death was again a major target identified by this analysis (Supplementary Figure 3). This was mirrored in the IFN-gamma treated fibroblast cells.

In order to look more closely at the DNA motifs identified from the ChIP-seq, we analysed gene ontology associated with these motifs using GOMO [39]. GOMO scores the upstream promoter of coding regions in the human database according to its binding affinity for the motif and attaches the gene ontology term to these putative targets of the motif. GOMO returns a significance value (p-value) for each GO term associated with a particular motif. Table 1 displays top predictions for Motifs 1-4 identified in the MEME-ChIP analysis (Figure 5 and Supplementary Figure 2). Some of the predictions overlapped or were present in each of the GOMO analyses for Motifs 1-4 such as antigen presentation, innate and adaptive immune responses and thus were not noted, as they do not distinguish the motifs from one another. As cell death is a major target pathway identified in our ChIP-seq experiment we wanted to identify any motifs that may be associated with this term. GOMO only returned two gene ontology terms which contained a number of cell death/apoptotic genes, this is ‘apoptotic protease activator activity (Motif 3 and 4) and ‘tumor necrosis factor receptor binding’ which contains a number of death associated genes such as TRAIL (Motif 1 and 4). Other interesting GO terms are ISG-15 protein conjugation associated with Motif 1 and 4, inflammatory response associated with Motif 2 and 4, DNA damage checkpoint associated with Motif 3 and 5, and the JAK-STAT cascade associated with Motif 2.

## Discussion

We undertook a high-throughput study to identify IRF1 binding sites in breast cancer cells in order to comprehensively identify target pathways that may contribute to IRF1’s tumor suppressor activity. IRF1 has been shown in numerous studies to inhibit the growth of transformed cells, in vivo and in vitro [40-44] but data in the field is still lacking in terms of the overall picture of its’ activities in this respect. Individual studies have identified specific apoptotic gene targets but whether these make up the majority of IRF1’s tumor suppressor activity is still unknown. Our ChIP-seq data indicates that the major tumor suppressor activity of IRF1 is the regulation of cell death pathways. We found that IRF1 binds to a large number of regions associated with apoptotic or cell death genes in both unstimulated and IFN-gamma stimulated cells. This was more apparent in the IFN-stimulated cells with 120 genes mapped to the cell death pathway. Our analysis of transcriptomic data in the GEO database supports the idea that IRF1 may activate these genes. In particular, apoptotic targets of IRF1 from the up-regulated transcripts were overwhelmingly associated with positive regulation of apoptosis (>80%).

We have found that IRF1 bound targets in IFN-stimulated cells are more likely to be up-regulated. Thus, IFN signaling may contribute to the ability of IRF1 to induce target genes. We hypothesize that this may be due to a number of reasons such as cis-acting factors working in cooperation with IRF1 in IFN-stimulated cells, chromatin environment in control vs. IFN-stimulated cells or modification of IRF1 in IFN-stimulated cells. Also, plotting of the number of IRF1 peak regions near transcriptional start sites increases after IFN stimulation and apoptotic targets are enriched in this pool of putative targets. Binding of transcription factors near the TSS indicates in many cases that there will be a direct affect on its transcription [45,46]. Thus many of these binding events where IRF1 is binding close to the TSS have an increased likelihood of being functional.

**Figure 3:**
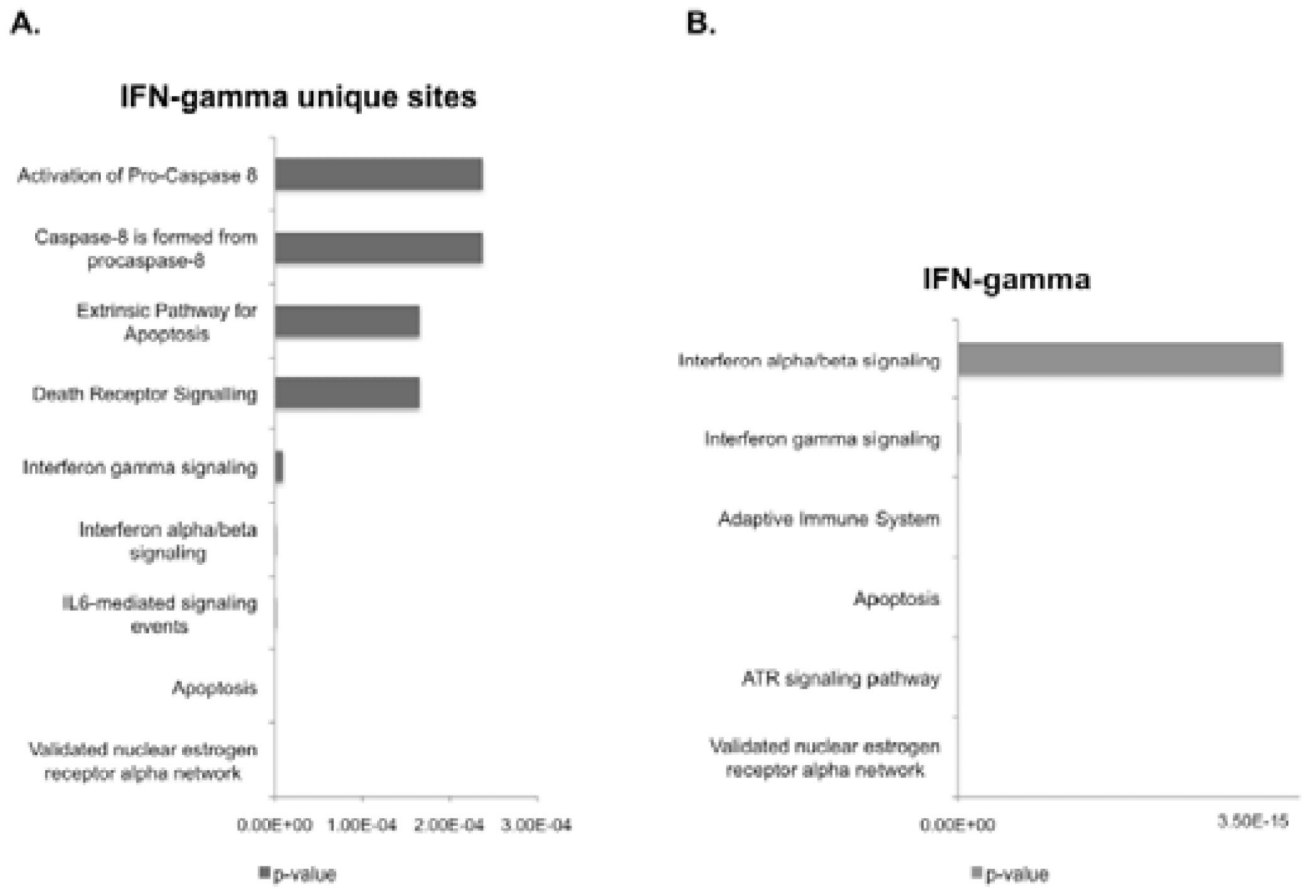
Functional categorization (Pathway Commons, GREAT) of peak regions from IFN-gamma stimulated cells A. The graph displays significantly enriched pathways associated with peak regions in IFN-gamma stimulated cells only (shown as p-value) B. The graph displays significantly enriched pathways associated with peak regions from all IFN-gamma binding events (shown as p-value).

**Figure 4:**
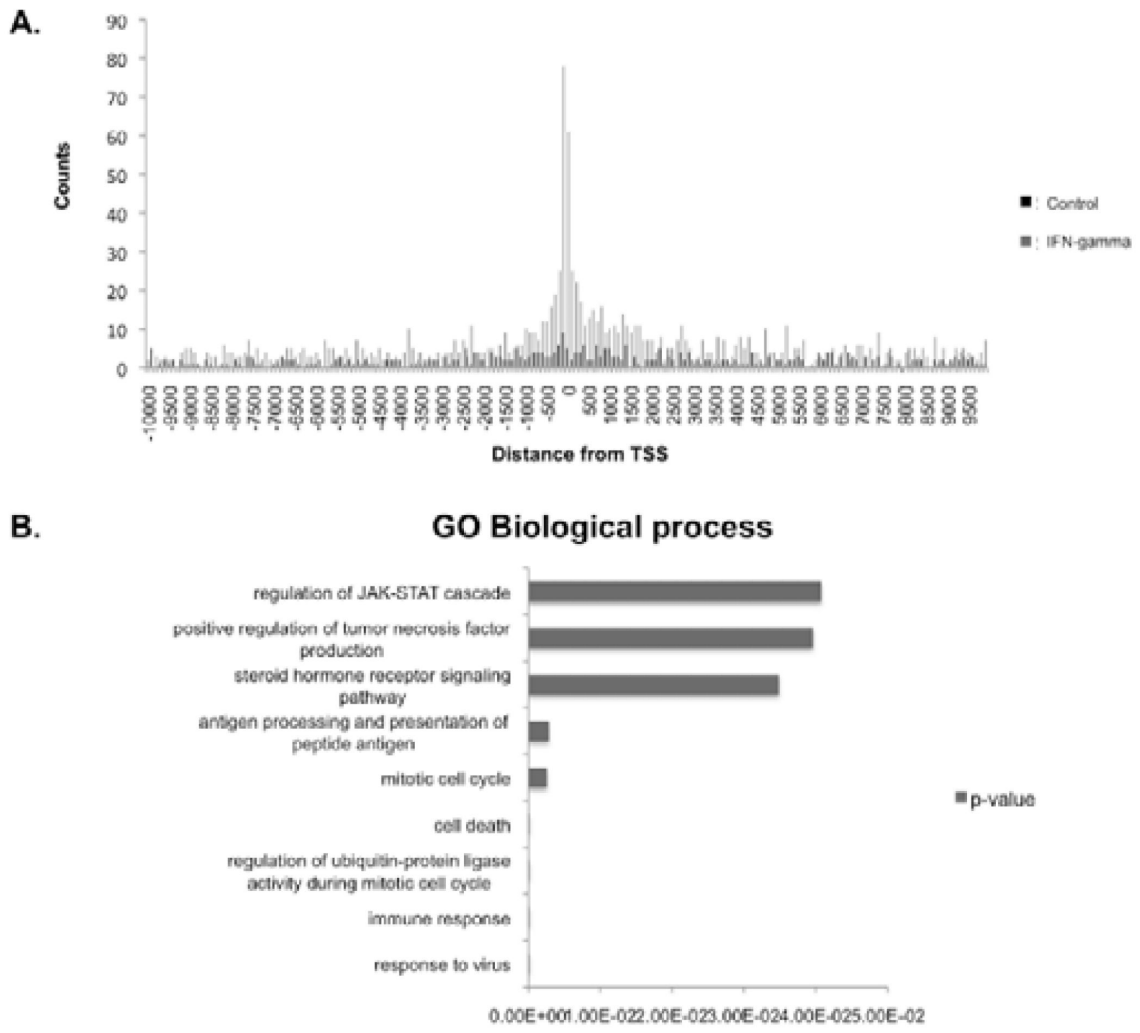
IRF1 peak regions are clustered around the transcriptional start sites (TSS) of genes in IFN-gamma stimulated cells A. The graph depicts the peak region counts versus distance from the TSS of a coding region (GPAT) for control (unstimulated) and IFN-gamma stimulated cells B. Shown are enriched biological processes for peak regions from IFN-gamma stimulated cells centred around 1 kb to 2 kb from the TSS. Graph shows the corresponding p-value.

**Figure 5:**
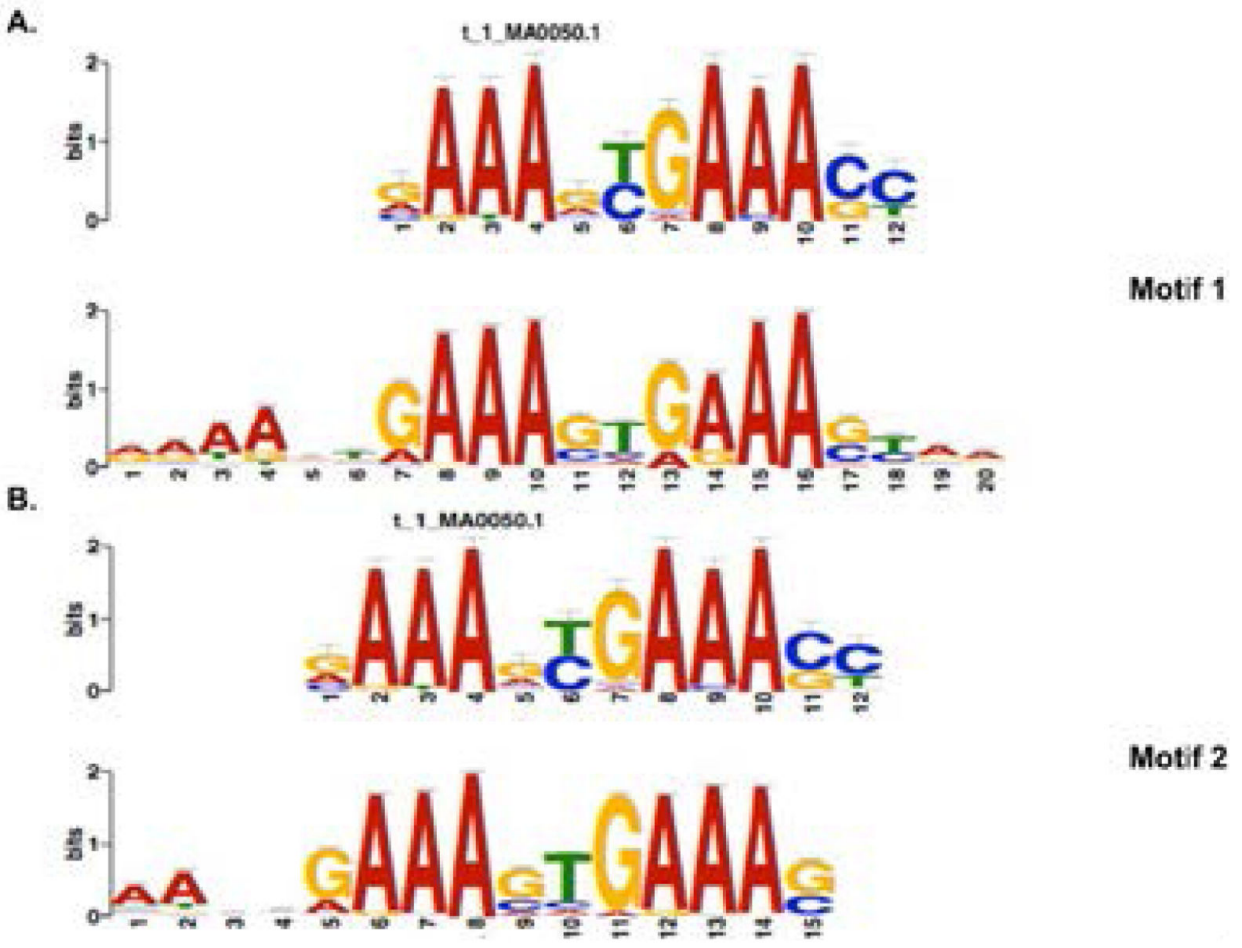
De novo motif discovery carried out on IFN-gamma stimulated peak regions reveals new determinants for IRF1 binding A. Shown is the logo for Motif 1 which is the most statistically significant DNA motif derived from all peak regions found in IFN-gamma stimulated cells (e-value, 1.2e-168) B. Shown is the logo for Motif 2 which is the most statistically significant DNA motif derived from IFN-gamma unique peak regions (only found in IFN-gamma stimulated cells)(e-value, 7.0e-455). Both motifs are aligned to the consensus IRF1 motif (MEME-ChIP).

**Figure 6:**
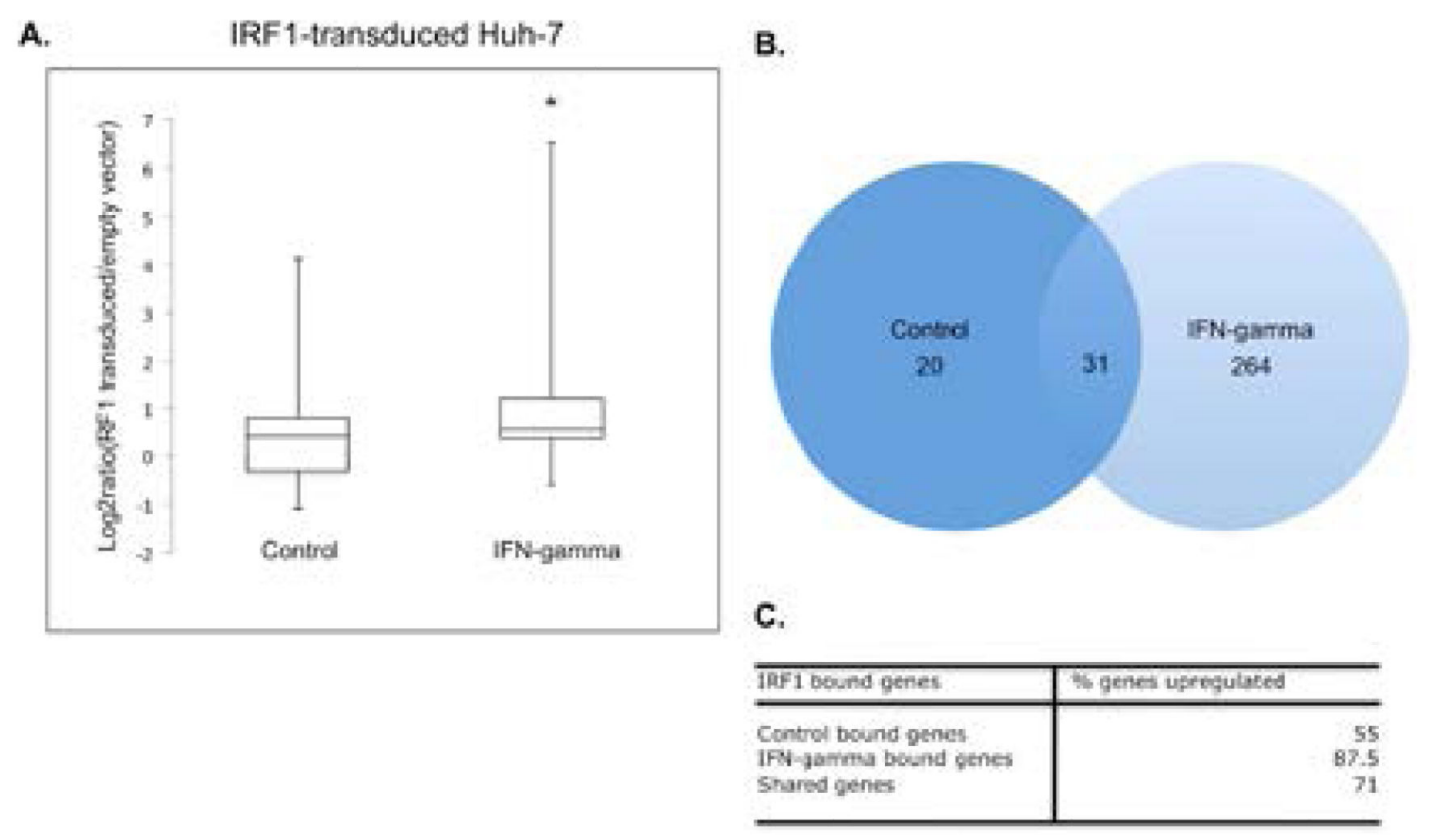
Gene expression analysis shows IRF1 bound genes in IFN-gamma stimulated cells are generally up-regulated A. Shown is the distribution of expression values (log_2_ ratios) from IRF1-transduced Huh-7 cells (GEO, GSE26817). Boxplots depict median value for the ChIP-seq control IRF1 bound gene group and IFN-gamma IRF1 bound gene group. Statistical difference between the two groups was evaluated using the Student t-test, *p-value, 0.0173) B. Venn diagram of total mapped genes from each ChIP-seq condition (control or IFN-gamma) present on the list of valid transformed log_2_ ratios (p-value ≤ 0.05) C. Shown is a table depicting the percent up-regulation of ChIP-seq bound genes from each condition, including overlapping genes.

A significant portion of the peak regions mapping to apoptotic genes are unique to IFN-stimulated cells and it may be that these genes only require the recruitment of IRF1 to their regulatory domains in IFN-stimulated cells to induce expression. A number of these genes map to the extrinsic cell death pathway and we found that this was indeed a significantly enriched GO biological process found uniquely in IFN-gamma stimulated cells (Figure 3). TRAIL, an IRF1 target, is bound only in IFN stimulated cells and is part of the extrinsic cell death pathway. Motif identification of the peak regions associated with apoptotic genes showed a match to the consensus IRF-E and strengthens the idea that these are direct targets of IRF1. Indirect binding of IRF1 to chromatin regions may occur in control (unstimulated) cells where we could not identify an enriched motif matching the IRF-E in this set of peak regions. We did identify other enriched motifs and these may be bound by IRF1 in unstimulated cells. Two other motifs were identified that associate with a gene ontology term containing genes involved in cell death (tumor necrosis factor receptor binding), which suggests that IRF1 may uniquely bind to these motifs to regulate the extrinsic death pathway. Three of the annotated genes FADD, TRAIL and caspase-8 were in this category and bound by IRF1 only in IFN-gamma stimulated cells.

Another interesting putative target pathway identified in IFN-gamma stimulated cells is the ATR signaling pathway. This pathway is important for the DNA damage response. A ChIP-chip study determined that IRF1 potentially regulates the Fanconi anemia pathway via its regulation of BRIP1 protein expression. Interestingly, the fanconi anemia pathway interacts with the ATR signaling pathway [47-49]. A number of other putative gene targets involved in the DNA damage response were identified in the ChIP-chip study and this ChIP-seq study adds to the number of genes that may contribute to IRF1’s role in the DNA damage response.

A large number of the putative target genes are categorized under immune or interferon responses. As IRF1 is an interferon-regulated gene and has been shown to bind interferon-stimulated genes, it indicates that the ChIP-sequencing was successful in finding bona fide IRF1 targets. The presence of the core consensus IRF-E within all the DNA motifs overrepresented in our target set also strengthens this conclusion. We found that all of the longer motifs (15-20bp) were associated with the promoter regions of genes involved in T-helper cell differentiation. This is important as IRF1 is known to be essential for proper differentiation of naïve T cells into Th1 cells [50]. DNA damage checkpoint genes were associated with Motif 3 and 5. These two motifs may constitute the binding site for IRF1 to regulate genes in the ATR signaling pathway identified as a putative new target pathway in IFN-gamma stimulated cells. Motif 2 is interesting as it is found in promoters of genes associated with the JAK-STAT cascade. IRF1 is involved in this pathway as it a downstream target of STAT1 dimers after IFN-gamma stimulation of cells. The JAK-STAT cascade was only associated with this motif suggesting that specific IRF1 binding motifs can elicit specific responses.

IRF1 inhibits cancer cell growth. Our study has added to our understanding of pathways regulated by IRF1 after IFN stimulation. Many of the putative target genes fall under processes known to be regulated by IRF1 such as apoptosis and DNA damage. Here, we have shown that IRF1 binding correlates with increased expression of genes in IFN-stimulated cells or IRF1-transduced cells. Expression targets are enriched in apoptotic genes which is the most over-represented biological process associated with IRF1 binding. These data indicate that the major anti-cancer pathway regulated by IRF1 is cell death and solidifies IRF1’s role as a tumor suppressor.

## Supplementary Material

Supplementary Data

Supplementary Table 1

Supplementary Table 2

Supplementary Table 3

Supplementary Table 4

## Figures and Tables

**Table 1:** Gene ontology analysis for overrepresented Motifs in peak regions.

Motif	Gene ontology (GO) term	Category	*p-value
**Motif 1**	Tumor necrosis factor receptor binding	MF	2.883e-04
	ISG-15 protein conjugation	BP	2.968e-04
	T-helper cell differentiation	BP	8.479e-06
	Transcription factor TFIIA complex	CC	4.663e-04
**Motif 2**	T-helper cell differentiation	BP	1.696e-05
	Inflammatory response	BP	2.544e-05
	JAK-STAT cascade	BP	4.918e-04
	Regulation of cell killing	BP	1.441e-04
**Motif 3**	Apoptotic protease activator activity	MF	1.865e-04
	DNA damage checkpoint	BP	4.324e-04
	mRNA metabolic process	BP	3.222e-04
	Regulation of chromosome organization	BP	2.544e-05
**Motif 4**	Inflammatory response	BP	5.935e-05
	Apoptotic protease activator activity	MF	4.494e-04
	Anti-apoptosis	BP	1.696e-04
	ISG-15 protein conjugation	BP	1.865e-04

* Statistical significance of associated gene ontology term.
