# Supplementary material for: Genome-wide Identification of IRF1 Binding Sites Reveals Extensive Occupancy at Cell Death Associated Genes
Source: J Carcinog Mutagen. Author manuscript; Available in PMC 2015 Apr 16. (PMC4398980; doi:10.4172/2157-2518.S6-009)
Supplement: Supplementary Data [file NIHMS62608-supplement-Supplementary_Data.pdf]

Supplementary Figure Legends

Supplementary Figure 1. Biological processes of control peak regions. Shown is a graph depicting the p-values of GO biological processes overrepresented in peak-associated genes from control (unstimulated) cells.

Supplementary Figure 2. Enriched motifs from IFN-gamma stimulated cells. Shown are logos from enriched motifs derived from Motif discovery using MEME-ChIP with corresponding e-values (statistical significance).

Supplementary Figure 3. GO biological processes of putative IRF1 gene targets from IRF1 transduced Huh-7 cells. Shown is a graph depicting the p-values of overrepresented GO-biological processes .

Supplementary Table 1. Excel list of annotated genes from the IRF1 ChIP-seq experiment. Shown is the count reads for each ChIP-seq condition, mock (no antibody), control IRF1 antibody, IFN-gamma IRF1 antibody. Chromosomal postions (start-min_pos and end-max_pos) are shown for each peak region, followed by the gene ID (Refseq), Gene symbol, strand, start position of the gene and distance of peak region from TSS of the corresponding gene.

Supplementary Table 2. Excel list of transformed expression data from GSE26817. Shown is the GEO2R transformed log2ratios (adjusted p-value ≤ 0.05) for genes mapped to the GSE26817 experiment from the IFN-gamma stimulated peak region annotated gene list.

Supplementary Table 3. Excel list of transformed expression data from GSE26817. Shown is the GEO2R transformed log2ratios (adjusted p-value ≤ 0.05) for genes mapped to the GSE26817 experiment from the control unstimulated peak region annotated gene list.

Supplementary Table 4. Excel list of transformed expression data from GSE26817. Shown is the GEO2R transformed log2ratios (p-value ≤ 0.05) for genes mapped to the GSE26817 experiment from the IFN-gamma stimulated peak region annotated gene list.

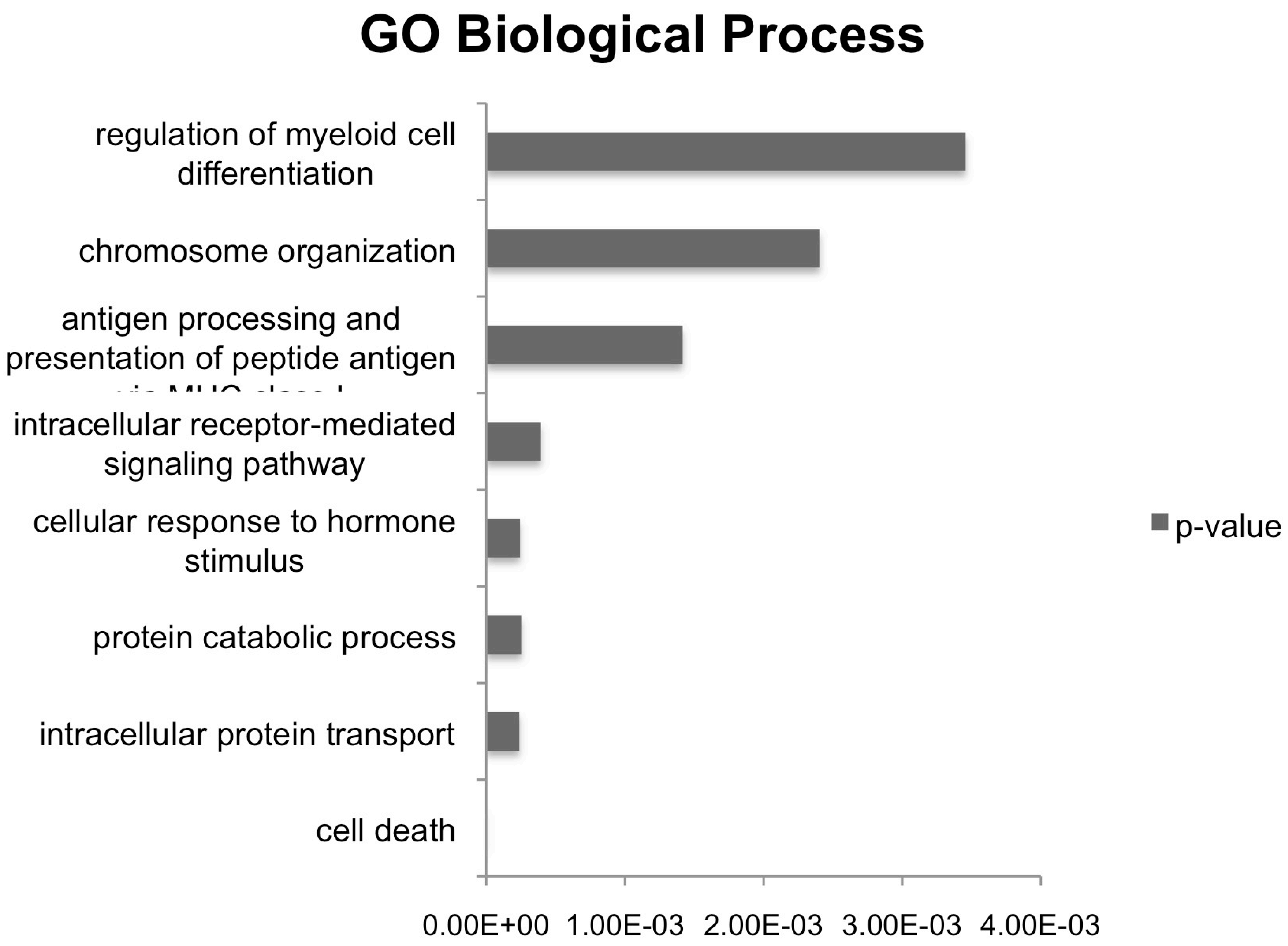

Supplementary Figure 1

A. IFN-gamma enriched motifs

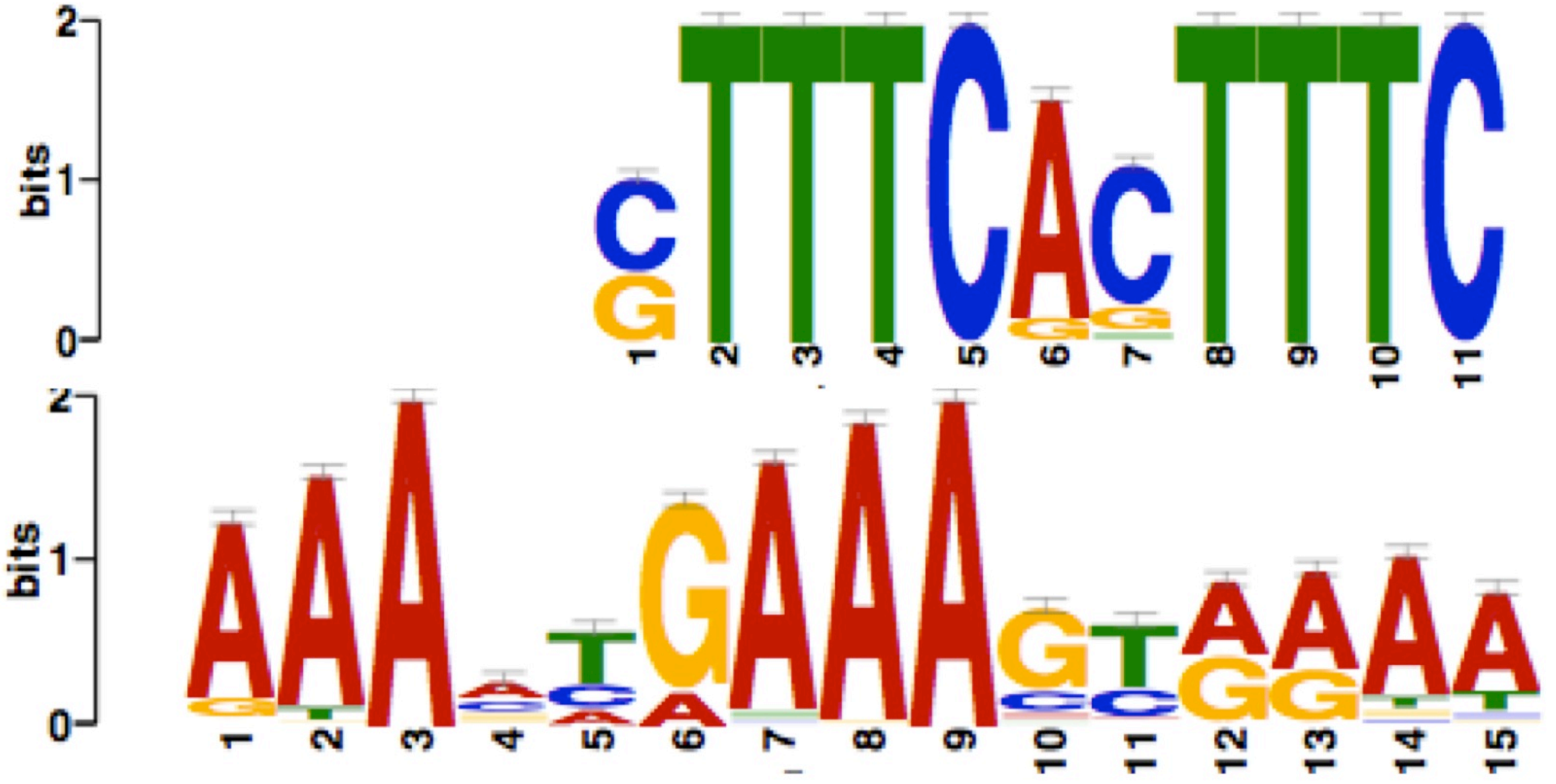

Motif	E-value
7	1.3e-81
5	8.3e-32

B. IFN-gamma unique enriched motifs

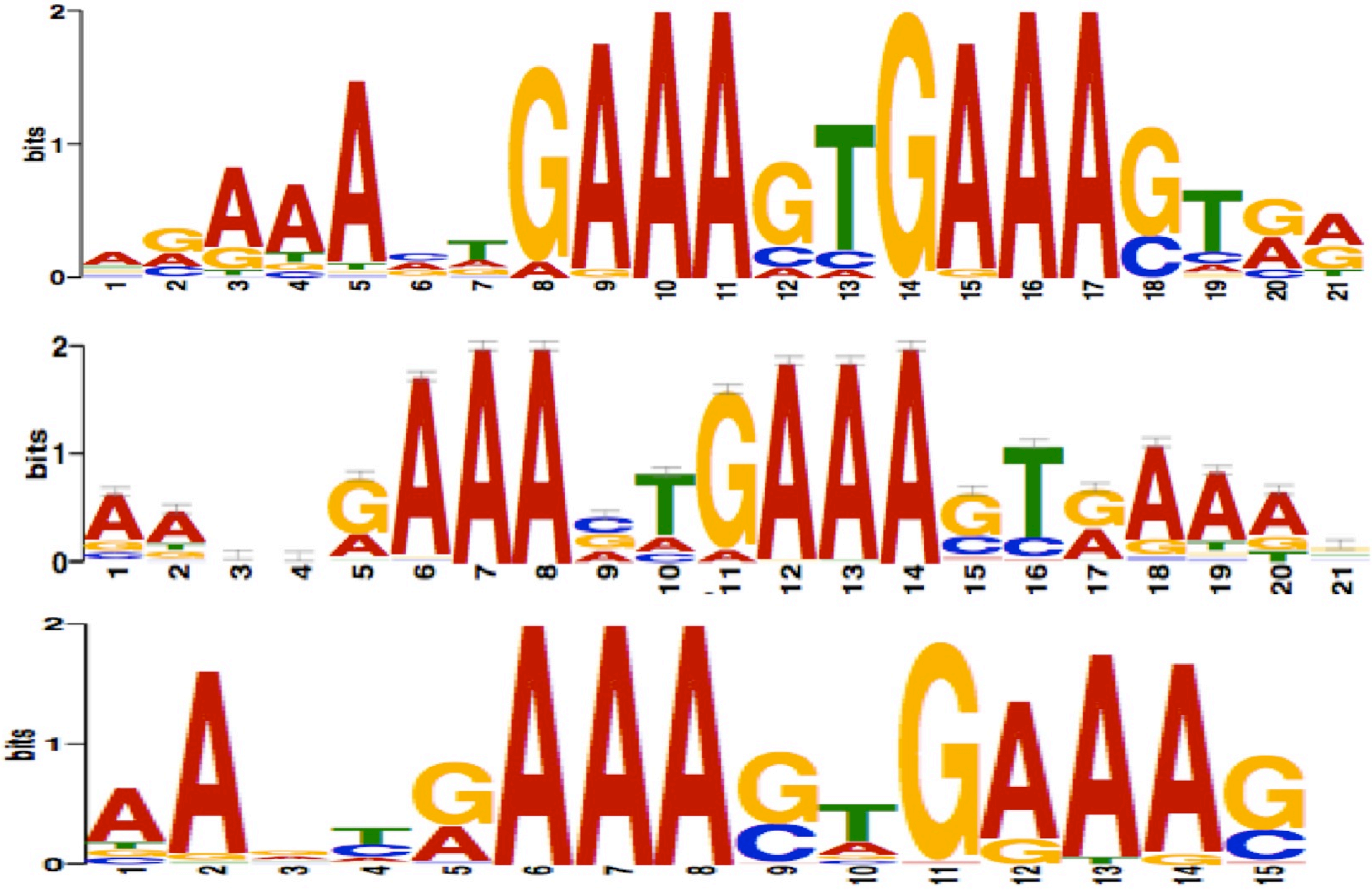

Motif	E-value
6	3.1e-124
3	1.1e-81
4	3.8e-56

Supplementary Figure 2

Go Biological process	Count	p-value
response to virus	14	9.34E-09
antigen processing and presentation of peptide antigen via MHC class I	7	1.18E-07
innate immune response	11	4.51E-05
JAK-STAT cascade	7	2.35E-05
negative regulation of protein modification process	10	7.82E-05
growth hormone receptor signaling pathway	4	1.79E-04
inflammatory response	15	4.16E-04
leukocyte activation	12	0.001121147
positive regulation of I-kappaB kinase/NF-kappaB cascade	7	0.003462038
hemopoiesis	10	0.009641889
regulation of apoptosis	22	0.009927461

Supplementary Figure 3
